# The Black Death

**Published:** 1921-10

**Authors:** A. T. Nankivell

**Affiliations:** (Medical Officer of Health, Hornsey)


### NOTABLE EPIDEMICS.

### I. THE BLACK DEATH.

By A. T. Nankivell, M.D., D.P.H. (Medical Officer of Health, Hornsey).

Towards the middle of the fourteenth century some Italian merchants who were occupied in trading with China by the overland route were besieged by the Tartars in a small town on the river Don. They were, however, driven out of this town, and many of them, together with their merchandise from China, found refuge in the fortified post of Caffa, in the Crimea. Later they were again besieged in this town by the Tartars, and the siege lasted for nearly three years. The Italian traders were, no doubt, greatly overcrowded at Caffa, and were exposed to all the hardships of a long siege. Plague broke out, either within the town or among the barbarians around it, and finally caused so great a mortality among the Tartars that the siege was raised, and the enemy returned home to spread plague throughout the Middle East. Those of the Italian merchants who were left alive departed by sea to Genoa, and although there was no plague on board their ship, the disease appeared at Genoa within a few days of their landing, in the spring of the year 1347. From Genoa the plague spread gradually over Europe, and it was not until 1350 that the infection had penetrated into Scotland and into the countries around the Baltic Sea.

### Plague in England.

Plague entered England at Weymouth in early August of the year 1348. It spread rapidly through Dorset, Somerset, and Devon. By August 15 Bristol was infected, and shortly afterwards Gloucester became invaded. The Black Death was in London before the end of the year, but the mortality did not reach its maximum until the spring of 1349. By March of that year the plague had spread to Norwich and the Eastern Counties, and a month or so later it had come to York. The spread northwards of the pestilence seems to have abated somewhat, and it does not appear to have become epidemic in Scotland until the following year. Ireland also suffered heavily from the plague for a period of four months from August to December, but it is uncertain whether this took place in 1348, as the result of a direct infection from the Continent, or in 1349, as an importation from England.

The Black Death seems, from the various accounts of it by contemporary writers, to have been both pneumonic and bubonic plague. The disease was characterised by sudden swellings in different parts of the body, especially in the armpits, neck, or groins; by hæmorrhages into the skin; and sometimes by the vomiting of blood. The pestilence was swift in doing its work, for one day people were in high health and the next day dead and buried. Sometimes the attack was fatal within twelve hours, and almost always within four days. The plague was so infectious that those who touched the dead frequently sickened and died; and often it happened that penitent and confessor were borne together to the same grave.

The distribution of the mortality was various. Those of high rank were not greatly affected, but of the common people an incalculable number died, and very many of the clergy, nuns, and friars. The religious houses seem especially to have been severely visited by the Black Death, and many of them were closed and left deserted.

The records of mortality in those days were indifferently kept, and it is most difficult to arrive with any accuracy at an estimate of the number of those who died of the plague. Contemporary writers vary in their estimates from one-fifth to nine-tenths of the total population as the extent of the mortality, but it is probable that between half and two-thirds would be a more accurate estimation. In other words, the death-rate in England during the years of the Black Death was somewhere near 600 per thousand persons living! It is probable that about 20,000 persons died in London, out of an estimated population of 40,000. In Leicester there were about 1,500 deaths among a population of under 4,000 persons. In Bodmin there were the same number of deaths out of a population of some 3,000 people. Norwich had, perhaps, 17,000 deaths out of a population which was probably about 25,000, although in the borough records the mortality was stated to be over 57,000, which is manifestly exaggerated. Yarmouth lost 7,000 of its 12,000 people. As far as we can read from the scarce records, mortality of a like nature was present through the length and breadth of the land. Business was dislocated; cattle and sheep were left to wander, for there was no one to care for them, and a murrain occurred among the beasts. As half the labour in the country was dead, wages rose to an unprecedented height, and, according to Creighton, many villeins and bondsmen took the opportunity of escaping to the towns or to distant manors, where they could make their own terms. The Black Death was, therefore, the last nail in the coffin of the old feudal system.

Although this epidemic of the plague came to an end in England in fourteen months or so, sporadic cases of the disease continued for many years afterwards, interspersed with occasional epidemics; and plague can be said to have been epidemic in England for more than 300 years. In 1361 the country suffered from a considerable outbreak, in which the mortality seems to have been peculiarly heavy among the well-to-do classes and among the children. Plague appeared on the continent of Europe two years before this second English epidemic, and it is said that in Florence hardly ten out of the 1,000 remained alive. Epidemics of plague broke out again in England in 1368 and in 1375, and there was a bad epidemic in 1390, when the incidence of mortality seems again to have been heavy among the

**Notable Epidemics—(*cont.*).**

children and young people. In the year 1405 there was another great outburst, and 30,000 persons are stated to have died in London; and subsequently, and until the time of the Great Plague in 1664, there were other epidemics, serious enough no doubt, but not notable in comparison with the Black Death. The people were becoming used to the presence of the disease; it was no longer something new and strange, and it is probable that both the attack-rate and the fatality diminished with succeeding epidemics as the population acquired a certain amount of immunity. By the middle of the fifteenth century the plague in England had no longer the terrible characteristics of a new disease in virgin soil.

Boccaccio, that teller of infinitely clever stories, gives an excellent account of the Black Death in Florence, and says in his preface to "The Decameron" that upwards of 100,000 persons perished in his city. "How many noble families became extinct!" he writes. "What riches, what vast fortunes, were left with no heir to inherit them! What numbers of both sexes in the prime of vigour and youth . . . after dining heartily with their friends here, have supped with their departed friends in another world!" A writer in Sienna says: "And I, Agniolo di Tura, carried with my own hands my five little sons to the grave; and what I did many others did likewise." Ships could be seen, laden with merchandise, driven hither and thither by the winds and seas, and all their crews dead of the pestilence. At Avignon, which at that time was the seat of the Pope Clement VI., 150,000 persons are said to have died: "Even the animals in the place, such as dogs, cats, cocks and hens, also died . . . Such men as had strength fled for fear, and in consequence many sick persons who might otherwise have recovered perished through want of care. Many, too, who were seized with the sickness, being considered certain to die and without any hope of recovery, were carried off at once to the pit and buried; and in this way many were buried alive." The mortality, says the physician of Clement VI., was of two kinds. "The first lasted two months with constant fever and blood-spitting, and of this people died in three days. The second lasted for the rest of the time (for five months more). In this, with constant fever, there were external carbuncles or buboes under the arm or in the groin, and the disease ran its course in five days. The contagion was so great—especially when there was blood-spitting—that not only by remaining [with the sick], but even by looking at them people seemed to take it; so much so that many died without any to serve them, and were buried without priests to pray over their graves. A father did not visit his son, nor the son his father. Charity was dead. The mortality was so great that it left hardly a fourth part of the population. . . Almost all those who were taken ill died, except towards the end of the epidemic, when some few recovered." In Styria it is related "often it happened that when one died in a house all, one after the other, were carried off. So certain was this that no one could be found to stay in the houses of the sick. . . As a result of this overwhelming visitation the cattle were left to wander in the fields without keepers, for none took any thought for the future; and the wolves coming down from the mountain to attack the cattle quickly fled into the wilds again, as if frightened by something unseen."

These extracts from very human documents give us some idea of the horror of those days.

### Some Epidemiological Questions.

Of the epidemiology of plague we know very little. Of the methods of its spread we know something. The pneumonic type is acutely infective, like measles or influenza, and the virus is probably carried from person to person by droplet infection. The bubonic type of plague is spread by the rat flea. But why plague arises (in China or in Caffa) we do not know, and we are ignorant of the reason for its disappearance. Plague, after being endemic in England for more than 300 years, became epidemic again in 1665, and then suddenly disappeared. No one can attribute the departure of the unwelcome guest to the sanitary reforms of those days, nor would it be allowable to infer that the people of our nation developed an absolute immunity to the *B. pestis*. It is no answer to the epidemiological problems to assert that plague bacilli, like the poor, are always with us, that they have existed as such since the early days of the earth, and that they alter in virulence to become either deadly or harmless. Such is no answer, unless, indeed, we can ascertain the causes that lead to this heightened or lowered virulence; and of these at the present time we have to confess that we are ignorant.

